# Nitric Oxide and Hydrogen Peroxide Mediate Wounding-Induced Freezing Tolerance through Modifications in Photosystem and Antioxidant System in Wheat

**DOI:** 10.3389/fpls.2017.01284

**Published:** 2017-07-19

**Authors:** Tong Si, Xiao Wang, Lin Wu, Chunzhao Zhao, Lini Zhang, Mei Huang, Jian Cai, Qin Zhou, Tingbo Dai, Jian-Kang Zhu, Dong Jiang

**Affiliations:** ^1^National Technique Innovation Center for Regional Wheat Production, Key Laboratory of Crop Physiology and Ecology in Southern China, Ministry of Agriculture, National Engineering and Technology Center for Information Agriculture, Nanjing Agricultural University Nanjing, China; ^2^Department of Horticulture and Landscape Architecture, Purdue University, West Lafayette IN, United States

**Keywords:** mechanical wounding, hydrogen peroxide, nitric oxide, signaling, wheat, freezing tolerance

## Abstract

Mechanical wounding is a common stress caused by herbivores or manual and natural manipulations, whereas its roles in acclimation response to a wide spectrum of abiotic stresses remain unclear. The present work showed that local mechanical wounding enhanced freezing tolerance in untreated systemic leaves of wheat plants (*Triticum aestivum* L.), and meanwhile the signal molecules hydrogen peroxide (H_2_O_2_) and nitric oxide (NO) were accumulated systemically. Pharmacological study showed that wounding-induced NO synthesis was substantially arrested by pretreatment with scavengers of reactive oxygen species and an inhibitor of NADPH oxidase (respiratory burst oxidase homolog, RBOH). On the contrary, wounding-induced H_2_O_2_ accumulation was not sensitive to NO synthetic inhibitors or scavenger, indicating that H_2_O_2_ acts upstream of NO in wounding signal transduction pathways. Cytochemical and vascular tissues localizations approved that RBOH-dependent H_2_O_2_ acts as long-distance signal in wounding response. Transcriptome analysis revealed that 279 genes were up-regulated in plants treated with wounding and freezing, but not in plants treated with freezing alone. Importantly, freezing- and wounding-induced genes were significantly enriched in the categories of “photosynthesis” and “signaling.” These results strongly supported that primary mechanical wounding can induce freezing tolerance in wheat through the systemic accumulation of NO and H_2_O_2_, and further modifications in photosystem and antioxidant system.

## Introduction

As one of the major abiotic stresses, freezing causes adverse effects on crop physiological and metabolic processes (Chinnusamy et al., 2007; Ahuja et al., 2010; Kosová et al., 2013). Freezing is also a factor that causes agronomic yield losses under global climate change (IPCC, 2007). The production of winter wheat (*Triticum aestivum* L.) can be considerably destroyed by untimely frost or cold spell in later spring (Asseng et al., 2011; Han et al., 2013). As yet, the effects of freezing stress on crop production and mechanisms of freezing tolerance have been widely studied (Zhong et al., 2008; Kane et al., 2013; Trischuk et al., 2014; Jurczyk et al., 2015). However, the strategies of alleviating the negative effects of freezing stress and the potential mechanisms are still far from clear.

Hydrogen peroxide (H_2_O_2_), one of the reactive oxygen species (ROS), acts as a central signal molecule that mediates plant abiotic resistance by enhancing the transcription of stress-responsive genes and antioxidant capacity especially in response to freezing stress (Neill et al., 2002; Torres and Dangl, 2005; Li et al., 2014a). H_2_O_2_ has also been implicated in the perception and transduction of stimuli signals to mediate plant physiological processes directly through long-distance signaling or indirectly through NADPH oxidase (respiratory burst oxidase homolog, RBOH) in various species (Miller et al., 2009; Xia et al., 2015; Evans et al., 2016). In recent years, nitric oxide (NO) has drawn many attentions due to its integral role in response to various abiotic stresses such as freezing, wounding, salinity, metal toxicity, and UV (Zhao et al., 2004; Shi et al., 2005; Lin et al., 2011; Puyaubert and Baudouin, 2014; Sun et al., 2014). The cross-talk between H_2_O_2_ and NO in response to abiotic or biotic stresses is still in debate. NO causes rapid H_2_O_2_ accumulation when exposing plants to herbivore attacks, ozone or *Tobacco Mosaic Virus* infection, pointing out that NO acts upstream of H_2_O_2_ (Orozco-Cárdenas and Ryan, 2002; Pasqualini et al., 2009; Liao et al., 2013). Reversely, H_2_O_2_ regulates NO in response to abscisic acid (ABA) induced stomatal closure and aphids infestation (Bright et al., 2006; Mai et al., 2014). A deep investigation of the relationship between H_2_O_2_ and NO thus becomes a very fruitful avenue to uncover the underlying mechanisms that plants respond to various abiotic stresses.

Systemic acquired acclimation (SAA) has emerged as a protection mechanism against various abiotic stimuli, including heat, cold, high light, UV, and salinity in plants (Rossel et al., 2007; Mittler and Blumwald, 2015; Gilroy et al., 2016). Calcium waves, hydraulic waves, and electric signals are supposed to be early signals involved in this response. SAA, which is accompanied by a long-distance signaling response, cannot be induced without the participation of H_2_O_2_ and NO (Alvarez et al., 1998; Jih et al., 2003; Arasimowicz et al., 2009). Mechanical wounding, which is induced by biotic (e.g., herbivore attack and pathogens infection) and abiotic (e.g., raining, wind, touching, and hailing) factors in plants, exists widely in nature. Wounding induces numerous abiotic stress responses in untreated leaves or tissues involved in SAA (Schilmiller and Howe, 2005; Capiati et al., 2006; Walley et al., 2007; Mousavi et al., 2013). H_2_O_2_ and NO participate as pivotal signal messengers in response to wounding (Orozco-Cárdenas and Ryan, 1999; Orozco-Cárdenas et al., 2001; París et al., 2007; Minibayeva et al., 2009), whereas their roles in wounding-induced adaptation responses to abiotic stresses are barely reported in the literatures. In consideration of the roles of H_2_O_2_ and NO in wounding response and the alleviation effects on freezing stress, it would be of great interest to investigate whether H_2_O_2_ and NO are pivotal hubs involved in wounding-induced freezing tolerance in untreated systemic leaves.

In our previous study, we have shown that mechanical stimulation is associated with cold responses in wheat by modifications in the chloroplast antioxidant system and proteome changes under field condition (Li et al., 2015). However, its in-depth mechanism remains obscure. This study was designed to investigate the mechanisms underlying the alleviation effects of mechanical wounding on freezing stress in untreated systemic leaves of wheat plants as well as the signaling transduction of wounding-induced H_2_O_2_ and NO between these leaves.

## Materials and Methods

### Plant Materials

Uniform seeds of winter wheat (*Triticum aestivum* L. cv. Yangmai 16) were selected. Seeds were surface-sterilized using 2.5% sodium hypochlorite for 10 min, rinsed five times with sterile distilled water and germinated in vermiculite. When the second leaf fully expanded, plants were transferred to plastic containers (45 cm in length, 35 cm in width, and 18 cm in height) with Hoagland’s nutrient solution for hydroponic culture with the following experimental conditions: air temperature of 25°C/18°C (day/night), a photoperiod of 14/10 h (day/night), a constant humidity of 80–90% and a photosynthetic photon flux density (PPFD) of around 600 μmol m^-2^s^-1^. The solution was renewed daily and bubbled over the whole experimental period. Upon the appearance of the sixth fully expanded leaves (six-leaf stage), they were used for the experiment.

### Experimental Design

#### Responses of Wheat Plants to Freezing Stress after Mechanical Wounding

To determine the effect of mechanical wounding on resistance to freezing stress, the fourth leaves (we termed local leaves) from the bottom of seedlings at the six-leaf stage were wounded according to the methods of Capiati et al. (2006) and Gaupels et al. (2016) with appropriate modifications. The main vein in the center of the fourth leaves was crushed five times with a hemostat and the interval of each injury was 10 mm. The strength of the crushing was made uniformly to each plant. Wounding did not cause any visible alterations such as necrosis around the injuries. Both wounded and unwounded plants were then divided into two equal groups for normal or low temperature treatments after 12 h of the wounding events. The freezing stress was conducted in the climate chamber at -2°C, while the normal temperature treatment was conducted in another chamber at 25°C. The photoperiod was set at 12/12 h (day/night) and PPFD was set at 600 μmol m^-2^s^-1^ during the day time.

The maximal photochemical efficiency of photosystem II (*F*v/*F*m), net photosynthetic rate (Pn), malondialdehyde (MDA) content and relative electrolyte conductivity (REC) were determined by the fifth leaves (we termed systemic leaves) as area of interest after 24 h freezing stress. In order to investigate the time-course of antioxidant system in response to mechanical wounding, the local leaves were prior wounded, then the local and systemic leaves of wounded and untreated plants were immediately harvested in liquid nitrogen at indicated times from 0 to 96 h to determine the activities of antioxidant enzymes.

#### Effect of H_2_O_2_ and NO on Freezing Tolerance Induced by Mechanical Wounding

To detect the involvement of H_2_O_2_ and NO in wounding-induced freezing tolerance, the systemic leaves were firstly foliar applied with 200 μM sodium nitroprusside (SNP, a NO donor), 200 μM N^G^-nitro-L-Arg methyl ester (L-NAME, an inhibitor of NOS-type enzyme), 200 μM tungstate (an inhibitor of NR), 200 μM 2-(4-Carboxyphenyl)-4,4,5,5-tetramethylimidazoline-1-oxyl 3-oxide (c-PTIO, a specific NO scavenger), 5 mM dimethylthiourea (DMTU, a H_2_O_2_ and OH⋅ scavenger) or 100 μM diphenyleneiodonium (DPI, a RBOH inhibitor). After 8 h full absorption, the local leaves were wounded as mentioned above. After a time interval of 12 h, one batch of the local and systemic leaves was collected and the activities of antioxidant enzymes as well as the expressions of their corresponding genes were analyzed. The other batch of the plants was exposed to freezing stress at -2 C for 24 h. *F*v/*F*m was then measured at 24 h on the systemic leaves. For time-course analysis of mechanical wounding and SNP induced changes in the tolerance to freezing stress, the local leaves were first treated with distilled water, 200 μM SNP or wounded. Freezing was started at indicated times by challenged at -2°C as mentioned above after water, SNP or wounding treatment. *F*v/*F*m was then determined by the systemic leaves after 24 h freezing stress.

#### The Relation of H_2_O_2_ and NO, and Their Sources in Response to Mechanical Wounding

To investigate the interrelation of wounding-induced H_2_O_2_ and NO, the local leaves were pre-treated with 200 μM L-NAME, 200 μM tungstate, 200 μM c-PTIO, 5 mM DMTU, or 100 μM DPI. Eight hours after spraying, the local leaves were wounded as described previously. Three hours later, the local and systemic leaves were used for cytochemical and histochemical detection or chemical quantification of H_2_O_2_ and NO. In addition, the local and systemic of wounded leaves as well as their corresponding control were collected at indicated times from 0 to 96 h after wounding treatment and used for the chemical quantification of H_2_O_2_ and NO. In order to investigate the sources of H_2_O_2_ and NO in response to wounding, one batch of the wounded and control leaves was collected at 3 and 18 h after wounding for analyzing the NR and NOS activities, while the other batch was harvested at 3 h for cytochemical detection of H_2_O_2_ and gene expression analysis of *RBOH*.

### Leaf Net Photosynthetic Rate and Chlorophyll Fluorescence

Net photosynthetic rate (Pn) was determined on systemic leaves after 1 h recovery after freezing treatment, using a portable photosynthesis system (LI-6400; LI-Cor, Lincoln, NE, United States). The air temperature, relative humidity, ambient CO_2_ concentration and photosynthetically active radiation (PAR) used in the measurement were 25°C, 85%, 380 μmol mol^-1^ and 1000 μmol m^-2^s^-1^, respectively. Chlorophyll fluorescence was detected by an imaging system (CF Imager; Technologica Ltd., United Kingdom) as described in our previous research (Li et al., 2014b). For the maximal photochemical efficiency of photosystem II (*F*v/*F*m), plants were dark-adapted for approximately 30 min before imaging and reading. *F*v/*F*m was imaged on systemic leaves with the whole leaf as area of interest and analyzed by the FluorImager software (Version 2.2; Technologica Ltd., United Kingdom).

### NO Detection and Quantification

Endogenous NO level was *in situ* detected by imaging the fluorescence of 4,5-diaminofluorescein diacetate (DAF-2DA, a highly specific molecular probe for NO) as described by Zhang et al. (2007). Wheat leaf squares (10 mm) were incubated in the buffer solution containing 10 μM DAF-2DA (prepared in 20 mM HEPES-NaOH, pH 7.4) and vacuum-infiltrated for 3 h in darkness at 25°C, and then washed thoroughly with the same buffer for 10 min to remove excess fluorophore. Finally, the leaves were visualized with a confocal laser scanning microscope system (CLSM; TCS-SP2; Leica Lasertechnik GmbH, Heidelberg, Germany) (excitation 495 nm; emission 515 nm). NO content was further determined by following the conversion of oxyhemoglobin (HbO_2_) to methemoglobin (MetHb) at 401 nm and 421 nm with a hemoglobin method as described by Pasqualini et al. (2009).

### ROS Histochemical Detection, Cytochemical Analysis, and Quantification

The histochemical staining of H_2_O_2_ production was monitored according to Thordal-Christensen et al. (1997) with some improvements. Leaf samples were immediately immersed in 1 mg mL^-1^ 3,3′-diaminobenzidine (DAB) solution (prepared in 50 mM Tris-acetate, pH 3.8), vacuum-infiltrated for 10 min and incubated for 12 h at 25°C under light. Then the leaves were bleached in 95% (v/v) boiled alcohol for 10 min to visualize the brown spots. After cooling down, photographs were taken using a microscope (IX71, Olympus Co., Tokyo, Japan). The $O_{2}^{–•}$ visual detection was monitored by the reaction of $O_{2}^{–•}$ and nitro blue tetrazolium (NBT) as originally described by Jabs et al. (1996). In total, the leaves were maintained in 2 mM NBT solution (50 mM Tris-HCl buffer, pH 6.1) and incubated at 25°C in dark. After 8 h fully reaction, the leaves were bleached in 95% (v/v) boiled alcohol for 10 min prior to photographed.

H_2_O_2_ was also detected by a confocal laser scanning microscope system (CLSM; TCS-SP2; Leica Lasertechnik GmbH, Heidelberg, Germany) as mentioned by Xia et al. (2011) with minor modifications. For the fully absorption of the chemicals in the whole plant, the roots of the seedlings were incubated overnight in the climate chamber at 25°C with 25 μM 2′,7′-dichlorofluorescein diacetate (H2DCF-DA), prepared in 20 mM HEPES-NaOH (pH 7.4). The leaves and sheaths were sliced 3 h after each treatment with a couple of blades. The cross-sections were then washed with the 20 mM HEPES-NaOH (pH 7.4) buffer for 20 min to remove excess fluorophore. Then, the cross-sections were transferred to glass slides mounted in glycerol:phosphate-buffered saline (1:1 v/v) and immediately examined with a CLSM (excitation 488 nm; emission 525 nm).

The cytochemical localization of H_2_O_2_ was conducted using the cerium trichloride (CeCl_3_) staining protocol according to Bestwick et al. (1997). The excised leaf tissue pieces (1–2 mm^2^) were incubated in freshly prepared 5 mM CeCl_3_ solution (50 mM 3-(*N*-mor-pholino) propanesulfonic acid (MOPS) buffer, pH 7.2) for 1 h. The leaf sections were then fixed in 1.25% (v/v) glutaraldehyde and 1.25% (v/v) paraformaldehyde buffer (50 mM sodium cacodylate, pH 7.2) for 8 h. After fixation, the tissues were washed twice in the same buffer and postfixed for 45 min in 1% (v/v) osmium tetroxide. Then the samples were dehydrated in a graded ethanol series (30–100%; v/v) and embedded in Eponaraldite (Agar Aids, Bishop’s Stortford, United Kingdom). After 12 h in pure resin, followed by a change of fresh resin for 4 h, the tissues were polymerized at 60°C for 48 h. The sections were then sectioned (70–90 nm) on a Reichert-Ultracut E microtome and mounted on uncoated copper grids (300 mesh). The blocks were ultimately examined at subcellular level using a transmission electron microscope (H7650; Hitachi, Tokyo, Japan) at an accelerating voltage of 80 kV.

The concentration of H_2_O_2_ was determined according to Willekens et al. (1997) by monitoring the absorbance of titanium peroxide complex at 410 nm. $O_{2}^{–•}$ production rate was measured according to Elstner and Heupel (1976) at an absorbance at 530 nm.

### Oxidative Damage Measurement

Leaf lipid peroxidation was determined by measuring the amount of MDA produced by the thiobarbituric acid (TCA) reaction as mentioned by Hodges et al. (1999). MDA content was calculated at 532 nm by subtracting the absorbance at 600 nm for non-MDA compounds. The electrolytic conductivity was conducted according to Griffith and Mclntyre (1993) with a conductivity bridge (DDS-307A, LEX Instruments Co., Ltd., China) before (C1) and after (C2) the leaves were boiled for 30 min. REC was calculated by the equation as: REC = C1/C2^∗^100%.

### NOS and NR Activity Assay

To assess NOS activity, the total protein was first measured as described by Jin et al. (2011). NOS activity was then measured after centrifugation at 13,000 × *g* for 20 min at 4°C as in González et al. (2012). Briefly, NOS activity was detected in 1 mL of reaction mixture containing 100 mM phosphate buffer (pH 7.0), 1 mM L-Arg, 2 mM MgCl_2_, 0.3 mM CaCl_2_, 4 μM BH_4_, 1 μM FAD, 1 μM flavin mononucleotide (FMN), 0.2 mM DTT, 0.2 mM NADPH and 200 μL of protein extract. The decrease in absorbance as a result of NADPH consumption was determined at 340 nm for 5 min. NOS activity was calculated using the extinction coefficient of NADPH.

The NR activity was assayed according to the method of Scheible et al. (1997) with some modifications. NR activity was performed by mixing one volume of extract with five volumes of 25°C prewarmed assay buffer containing 100 mM HEPES-NaOH (pH 7.5), 5 mM KNO_3_, and 0.25 mM NADH. The reaction was started by the adding assay buffer, incubated at 25°C for 30 min, and then stopped by adding 0.1 M zinc acetate. After 15 min, the tubes were centrifuged at 13,000 *g* for 10 min. The nitrite produced was measured at 520 nm by adding 1 mL of 1% (w/v) sulfanilamide in 3 M HCl plus 1 mL of 0.02% (v/v) *N*-(1-naphthyl) – ethylenediamine in distilled water.

### Antioxidant Enzyme Extraction and Activity Assay

Frozen leaf tissues (0.5 g FW) were ground in 5 mL ice-cold 25 mM HEPES-NaOH buffer (pH 7.8) containing 20% (v/v) glycerol, 1 mM EDTA, 1 mM ascorbic acid (AsA), 5 mM MgCl_2_, 1 mM reducing glutathione (GSH) and 1 mM DTT. The homogenates were centrifuged at 4°C for 20 min at 12,000 *g* and the resulting supernatants were collected for enzyme analysis. In addition, the protein content were determined as mentioned by Bradford (1976). Superoxide dismutase (SOD) activity was determined by measuring the ability to inhibit the photochemical reduction of NBT according to Stewart and Bewley (1980). One unit of SOD activity was defined as the amount of enzyme required to cause 50% inhibition of the reduction of NBT at 560 nm. Catalase (CAT) activity was assayed as a decrease at 240 nm following the method of Patra et al. (1978). Ascorbate peroxidase (APX) activity was performed by a decline at 290 nm as previously described (Nakano and Asada, 1981) with minor modifications. Glutathione reductase (GR) activity was modified depending on the rate of decrease in the absorbance of NADPH at 340 nm as originally described by Foyer and Halliwell (1976).

### Extraction of Total RNA and Semi-quantitative RT-PCR Analysis

Total RNA was isolated from wheat leaves using the Trizol reagent according to the procedure supplied by the manufacturer and dissolved in diethyl pyrocarbonate-treated water after extraction. The first-strand cDNA template for RT-PCR was synthesized from 2 μg of total RNA following the manufacturer’s instructions (Sangon, Shanghai, China). The RT-PCR were performed using 2 μL two-fold diluted cDNA, 10 pmol of each oligo nucleotide primer and 1 U of Taq polymerase (Takara, Dalian, China) in 25 μL reaction solution. The gene-specific primers were constructed based on their cDNA sequences and were used for amplification as listed in Supplementary Table S1. The optimum PCR cycle number was adjusted for each gene in preliminary experiments. The aliquots of PCR products were loaded and separated onto agarose gels (2%) stained with ethidium bromide.

### High-Throughput mRNA Sequencing Analysis

Four treatments of systemic leaves: control (ck), 3 h after wounding (wo), 24 h after freezing (fr) and 3 h wounding + 24 h freezing (wo_fr) were collected and total RNA was extracted as mentioned above for library construction and mRNA sequencing. The mRNA sequencing analysis was conducted in Shanghai Center for Plant Stress Biology, Chinese Academy of Sciences. Reads of RNA-seq data were mapped to wheat reference genome (*Triticum_aestivum.* TGACv1.30). Differentially expressed genes were defined based on the default of Cuffdiff (used for analyzing transcriptome assembly and differential expression of RNA-Seq in this paper). All results were average of three biological replicates. GO enrichment analysis was performed by AgriGO. The histogram was generated using R^1^. Raw Illumina sequences and assembled sequences are available in the Gene Expression Omnibus (GEO) database of the National Center for Biotechnology Information (NCBI) (accession number: SRP110839).

### Statistical Analyses

Data were statistically analyzed by the SPSS software (version 11.0; SPSS Inc., Chicago, IL, United States). One-way ANOVA was used on the data sets and tested for significant (*P* < 0.05 and *P* < 0.01) treatment differences using Tukey’s test.

## Results

### Wounding Induces Freezing Tolerance Systemically in Wheat Plants

**FIGURE 1:**
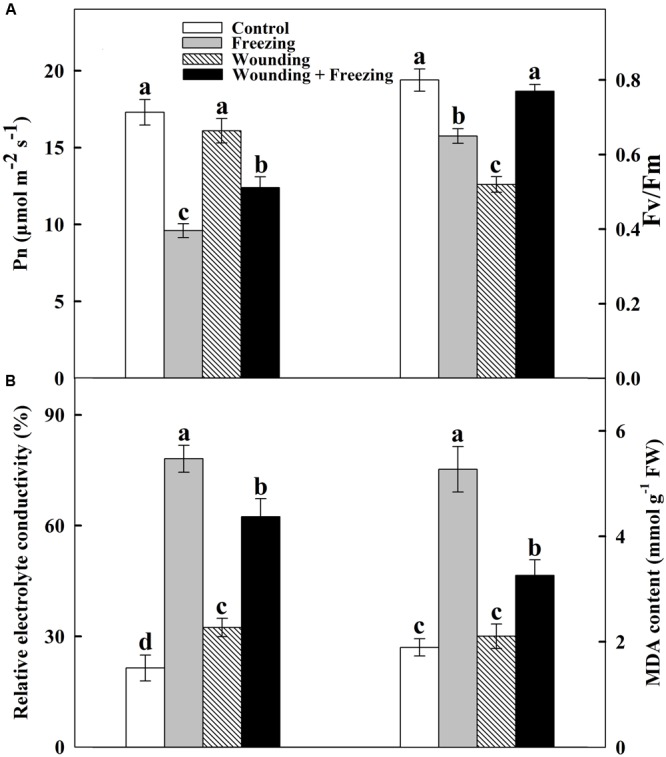
Effect of mechanical wounding of the local leaf on the maximal photochemical efficiency of photosystem II (*F*v/*F*m) and net photosynthetic rate (Pn), and the relative electrolyte conductivity (REC) and content of malondialdehyde (MDA) in the systemic leaf of wheat. The local (fourth) leaf was wounded 3 h prior to exposed to the freezing stress for 24 h. *F*v/*F*m and Pn of the systemic (fifth) leaf were determined as area of interest after freezing stress **(A)**. The systemic leaves were harvested after freezing stress for measurements of REC and MDA content **(B)**. Data are means ± SD of three different replicates. Means denoted by the same letter did not significantly differ at *P* < 0.05 for each analysis according to Tukey’s test.

Our first objective of this study was to determine whether mechanical wounding enhances freezing tolerance in wheat seedlings. Local leaves were prior wounded and the whole plants were further subjected to a 24 h freezing stress. Fluorescence images revealed that freezing treatment markedly decreased *F*v/*F*m in the fifth leaves from the bottom of the wheat plants (called systemic leaves) in unwounded plants compared to control (**Figure 1A**). Nevertheless, prior wounded plants sharply restored in *F*v/*F*m of systemic leaves in response to freezing stress. Besides, net photosynthetic rate (Pn) was determined after 24 h freezing treatment to assess the effect of mechanical wounding on freezing tolerance. Freezing stress caused a significant reduction in Pn in untreated plants. Local mechanical wounding alleviated freezing stress in systemic leaves accompanied by increased Pn compared to unwounded plants. In addition, mechanical wounding has no influence on Pn in wounded-only plants compared to that of control (**Figure 1A**).

We also examined the role of mechanical wounding in plant responses to freezing stress by comparing the REC and lipid peroxidation based on the MDA content after 24 h freezing treatment. REC and MDA content were remarkably increased by freezing stress in systemic leaves of unwounded plants whereas those in local wounded plants were declined but were still maintained in a relative high level compared to that of control and wounded-only plants (**Figure 1B**), indicating that mechanical wounding plays dominant roles in reducing membrane lipid peroxidation under freezing stress. These results revealed a novel role of local mechanical wounding on enhancing tolerance to freezing stress systemically in wheat plants.

### Involvement of H_2_O_2_ in Wounding-Induced NO Production

**FIGURE 2:**
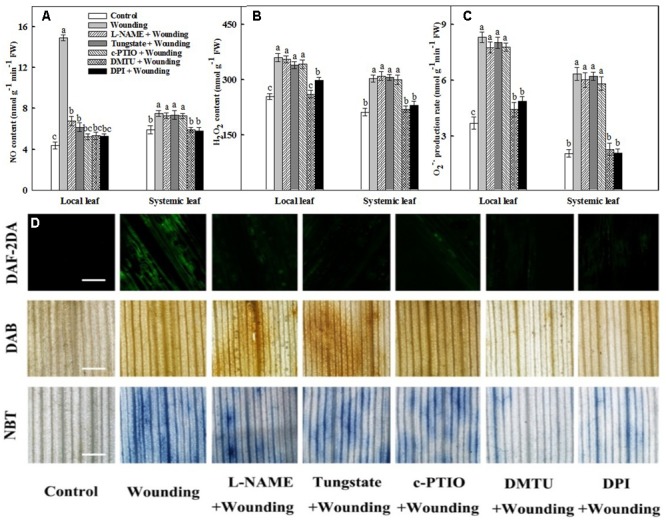
Mechanical wounding-induced the accumulation of NO, H_2_O_2_, and $O_{2}^{–•}$ in local leaf and systemic leaf. The local (fourth) leaf was pre-treated with distilled water, 200 μM L-NAME, 200 μM tungstate, 200 μM c-PTIO, 5 mM DMTU, or 100 μM DPI prior to wounding. The local leaves were harvested 3 h after wounding and the *in situ* NO was determined using DAF-2DA and detected by a confocal laser scanning microscope system (CLSM). The green fluoresce intensity of DAF-2DA indicates the degree of NO accumulation. Bar, 100 μm. H_2_O_2_ and $O_{2}^{–•}$ were determined in local leaf by incubating in DAB or NBT and photographed with a microscopy. Images of DAB and NBT stained leaves showing localized H_2_O_2_ and $O_{2}^{–•}$ accumulation as dark-brown and dark-blue spots, respectively. Bars, 2 mm. At least five leaves were imagined for each replicate and one representative is shown **(D)**. The other batch of local and systemic (fifth) leaves was also collected and NO concentration **(A)**, H_2_O_2_ content **(B)** as well as $O_{2}^{–•}$ production rate **(C)** were determined. Means denoted by the same letter did not significantly differ at *P* < 0.05 for each treatment according to Tukey’s test.

To detect the interaction of NO and H_2_O_2_ in response to mechanical wounding in wheat seedlings, we pre-treated the local leaves with L-NAME (an inhibitor of NOS), tungstate (an inhibitor of NR), c-PTIO (a specific NO scavenger), DPI (an inhibitor of RBOH), or DMTU (a H_2_O_2_ and OH⋅ scavenger) prior to wound treatment for 8 h. As shown in **Figures 2A,B**, pre-treatment of L-NAME, tungstate or c-PTIO almost completely blocked wounding-induced NO accumulation in local leaves. Again, pre-treatment of DMTU or DPI substantially reduced wounding-induced NO level. In contrast, pre-treatment of plants with DMTU or DPI thoroughly scavenged H_2_O_2_ and $O_{2}^{–•}$ in local leaves as expected. In reverse, pre-treatment of L-NAME, tungstate or c-PTIO had no effects on H_2_O_2_ content and $O_{2}^{–•}$ production rate (**Figures 2B–D**). Based on these observations, H_2_O_2_ appears to act upstream of NO in response to mechanical wounding. Namely, the generation of ROS including H_2_O_2_ and $O_{2}^{–•}$ is essential for wounding-induced the accumulation of NO.

### NO Is Involved in Wounding-Induced Freezing Tolerance

**FIGURE 3:**
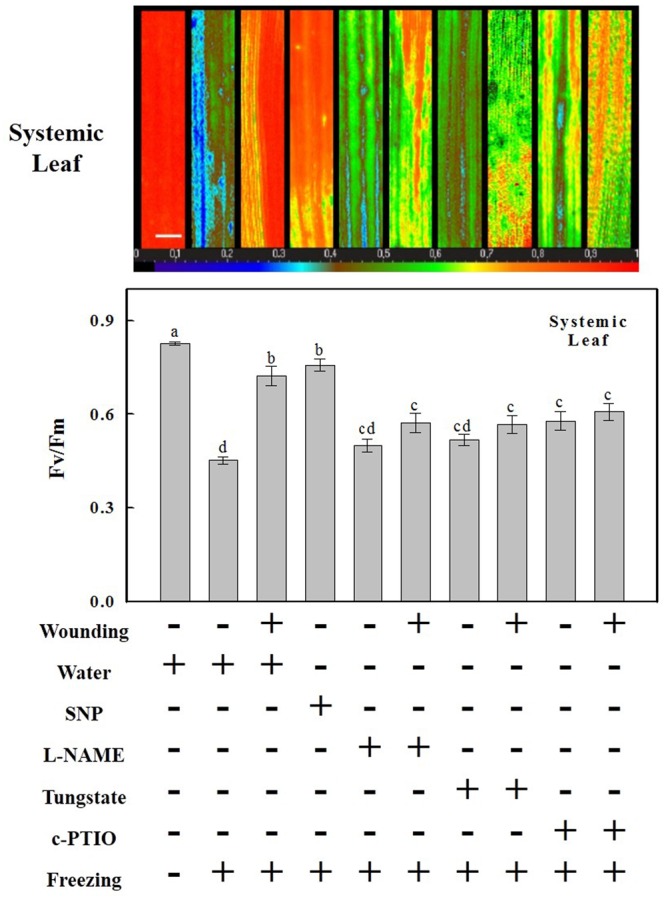
The involvement of NO in the maintenance of *F*v/*F*m of the system (fifth) leaf of cold stressed wheat plants by pre-wounding of the local (fourth) leaf. The systemic leaf was pre-treated with distilled water, 200 μM SNP, 200 μM L-NAME, 200 μM tungstate or 200 μM c-PTIO prior to wounded 8 h later. *F*v/*F*m was then determined by the systemic leaf as area of interest after 24 h freezing stress and one representative is shown. The horizontal false color code ranges from 0.0 (black) to 1.0 (red). Bar, 10 mm. Average values of *F*v/*F*m of systemic leaf by these treatments were also shown here. Data are means ± SD of three different replicates. Means denoted by the same letter did not significantly differ at *P* < 0.05 according to Tukey’s test.

Nitric oxide content was rapidly elevated systemically after wounding, suggesting that it may play a profound role in the subsequent freezing tolerance. To address this possibility, the NO donor SNP, synthesis inhibitors L-NAME, tungstate, and scavenger c-PTIO were applied to treat plants. The freezing tolerance was evaluated by measuring the reduction of *F*v/*F*m. Both wounding and SNP pre-treatment alleviated freezing-induced decline of *F*v/*F*m in systemic leaves (**Figure 3**). In plants pre-treated with L-NAME, tungstate or c-PTIO, however, the *F*v/*F*m was substantially decreased after freezing treatment. These results suggest that NO is essential for strengthening the photosynthetic system under freezing stress.

To further characterize the role of NO in wounding-induced freezing tolerance, the effect of freezing-mediated oxidative stress applied at different intervals after SNP or wounding treatment was determined. Enhanced tolerance of the systemic leaves to freezing-induced oxidative stress was observed as early as 3 h after treatment with SNP and 6 h after mechanical wounding, suggesting that exogenous NO induced freezing tolerance more rapidly than wounding. As depicted in Supplementary Figure S1, the maximum level of freezing tolerance was observed at 6 h after treatment with SNP and at 12 h after mechanical wounding, respectively. No significant level of freezing tolerance was observed at 48 h after treatment with SNP. Nevertheless, the alleviation effect of mechanical wounding still existed during the remainder of the time course, only slightly declined compared to that of 12 h (Supplementary Figure S1).

**FIGURE 4:**
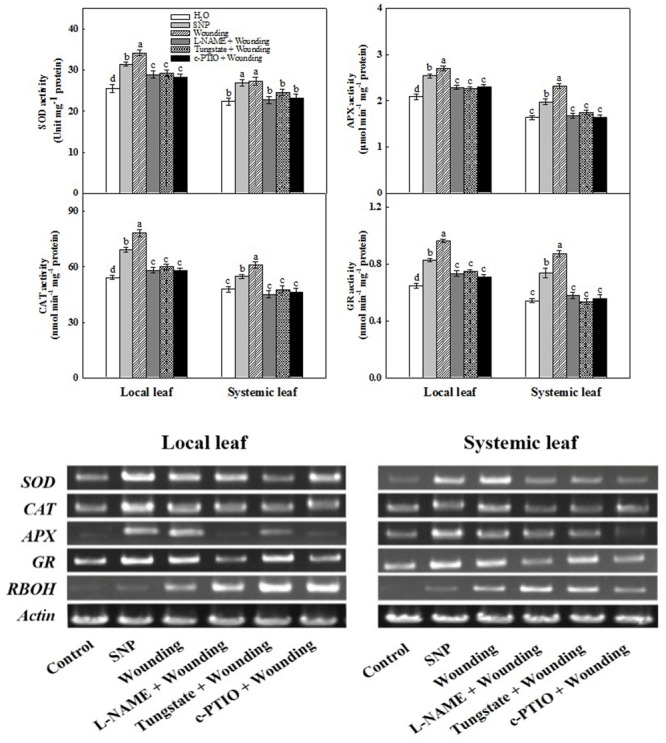
Requirement of NO in wounding-induced activities of antioxidant enzymes and the expression of their corresponding genes as well as *RBOH*. The systemic (fifth) leaf was pre-treated with distilled water, 200 μM SNP, 200 μM L-NAME, 200 μM tungstate or 200 μM c-PTIO prior to mechanical wounding. The local and systemic leaves were harvested 3 h after wounding for analysis the activities of antioxidant enzymes and the expression levels. Data are means ± SD of three different replicates. Means denoted by the same letter did not significantly differ at *P* < 0.05 for each leaf according to Tukey’s test.

We then determined the role of NO in the up-regulation of antioxidant defense systems after wounding or SNP treatment. Local mechanical wounding increased the activities of SOD, CAT, APX, and GR not only in local leaves but also in systemic leaves at 3 h in comparison with normally grown counterpart (**Figure 4**). The increased activities of these enzymes were effectively blocked by pre-treatments with L-NAME, tungstate or c-PTIO in both local and systemic leaves, suggesting that wounding-induced systemic tolerance is mediated by the signaling of NO. Analysis of the antioxidant-related gene expression showed that the *SOD, CAT, APX*, and *GR* transcription levels coincide with their corresponding enzyme activities. In particular, the expression of *RBOH* was profoundly higher in both local and systemic leaves after wounding, whilst NO donor as well as its inhibitors and scavenger had no significant effect on its expression (**Figure 4**). Collectively, we have clearly demonstrated that NO is involved in wounding-induced systemic freezing tolerance in wheat plants.

### Source of Endogenous NO and H_2_O_2_ in Response to Wounding

**FIGURE 5:**
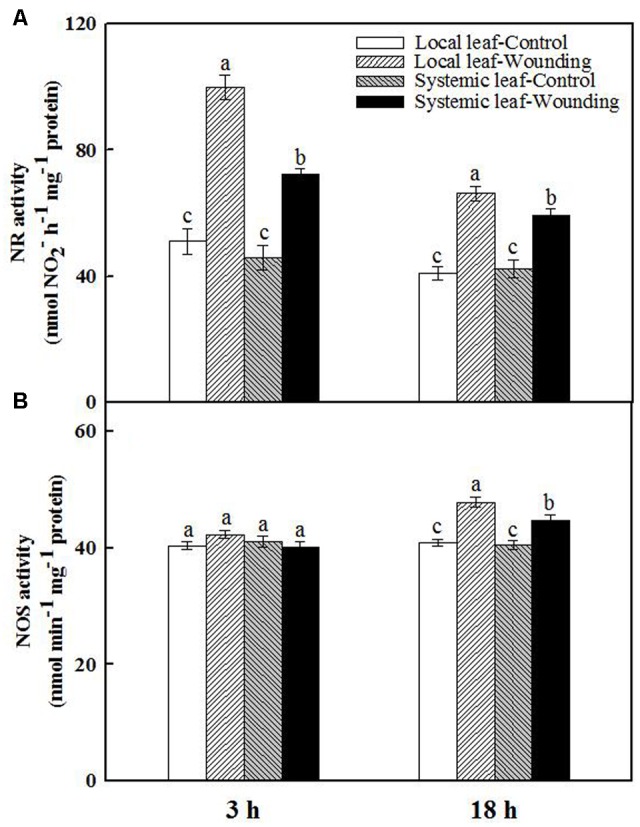
Effect of mechanical wounding on activities of nitrate reductase (NR) **(A)** and nitric oxide synthase (NOS) **(B)** in local (fourth) and systemic (fifth) leaves. The local leaves were wounded and then both the local and systemic leaves were collected for analyzing the activities of NR and NOS at 3 and 18 h after wounding, respectively. Data are means ± SD of three different replicates. Means denoted by the same letter did not significantly differ at *P* < 0.05 for each leaf according to Tukey’s test.

To elaborate the possible source of wounding-induced early NO burst in wheat plants, we analyzed NR and NOS activities in both wounded local leaves and unwounded systemic leaves. Mechanical wounding substantially elevated NR activity in both local and systemic leaves at 3 h, with 2.0- and 1.6-fold increase as compared with the untreated plants, respectively. The NR activity was attenuated at 18 h but still maintained at high levels compared to unwounded plants (**Figure 5A**). In contrast, no significant difference in NOS activity was observed between wounded and unwounded plants at 3 h after wounding (**Figure 5B**). However, the NOS activity in local and systemic leaves of wounded plants was 1.2- and 1.1-fold higher than that of controls at 18 h after wounding. These data imply that NR may mediate early NO burst while NOS is likely to act at a later phase in response to mechanical wounding.

**FIGURE 6:**
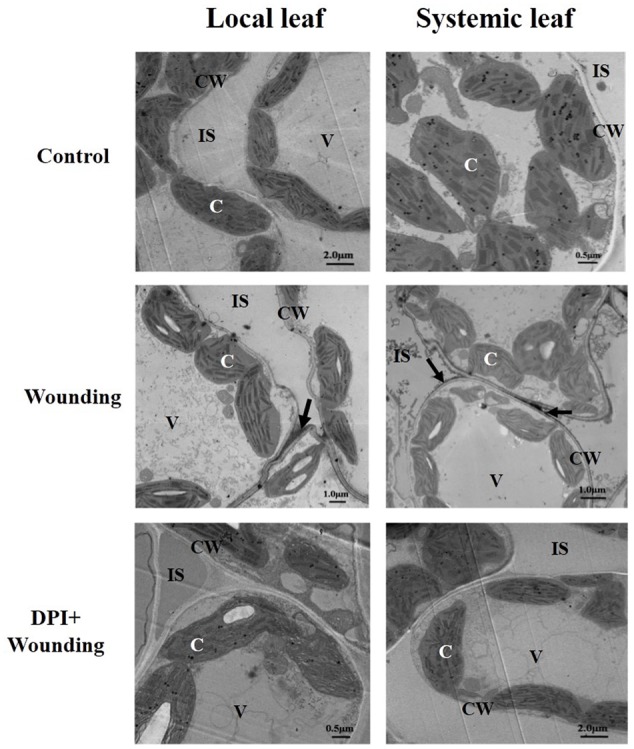
The cytochemical localization of H_2_O_2_ in local (fourth) and systemic (fifth) leaves. The local leaf was pre-treated with 100 μM DPI or distilled water as control. Mechanical wounding was conducted 8 h later. The 12 h after wounding, both the local and systemic leaves were collected for CeCl_3_ staining and detected by a transmission electron microscopy. Arrows, CeCl_3_ precipitates; C, chloroplast; CW, cell wall; IS, intercellular space; V, vacuole.

To further confirm the origination of H_2_O_2_ caused by mechanical wounding, samples were detected at cytochemical level with CeCl_3_ staining. As depicted in **Figure 6** and Supplementary Figure S2, H_2_O_2_ accumulation was predominantly distributed in the cell walls of local and systemic leaves but was only detected in chloroplasts of local leaf after 12 h mechanical wounding. In comparison, wounding-induced H_2_O_2_ accumulation was visible only in chloroplasts of local leaf in DPI- (an inhibitor of plasma membrane NADPH oxidase) treated plants (Supplementary Figure S2) and no CeCl_3_ staining was observed in the apoplast systemically (**Figure 6**). These results highlight the potential role of plasma membrane RBOH in the wounding signal transduction.

### Time Course of Wounding-Induced NO and H_2_O_2_ Accumulation, Systemic Tolerance, and Activities of Antioxidant Enzymes

**FIGURE 7:**
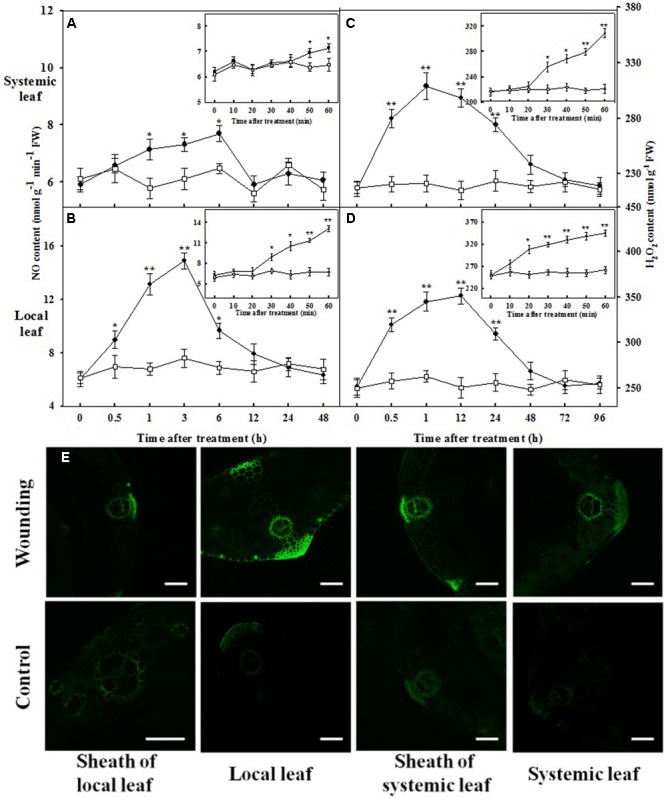
Kinetics of changes in NO content, H_2_O_2_ content and imagines of H_2_O_2_ localization in the vascular tissues in response to mechanical wounding. The local (fourth) leaf was wounded and then harvested at indicated times after wounding treatment. NO and H_2_O_2_ content of the local or systemic leaf of wounded (circles) and untreated (squares) plants were assayed. Time zero points represent without wounding treatment. The top and bottom graphs represent the systemic and local leaves, respectively **(A–D)**. Data are means ± SD of three different replicates. Insets: the average ± SE of two treatments in which the samples were harvested with an interval of 10 min for a better understanding of NO and H_2_O_2_ accumulation. Significance of variance for one-way ANOVA is indicated: ^∗^*P* < 0.05; ^∗∗^*P* < 0.01. **(E)** The roots of the whole seedlings were incubated into H2DCF-DA over night. The local leaf was prior wounded, after which the leaves and stems were sliced 3 h later and detected by a CLSM (excitation, 480 nm; emission, 530 nm). Bars, 100 μm.

To obtain insight into the inter-relationship between NO and H_2_O_2_, we also detected their local and systemic accumulation triggered by mechanical wounding. A highly transient increase of NO was observed after 30 min of mechanical wounding in local leaves, attained peak level at 3 h and remained elevated for at least 6 h (**Figure 7B**). The elevated NO content was also observed in systemic leaves at 50 min and remained significantly higher until returned to the resting level at 12 h after wounding treatment. Nevertheless, NO content was comparatively lower than that in local leaves (**Figure 7A**). The intercellular H_2_O_2_ displayed a continual increase as early as at 20 min in local leaves and at 30 min in systemic leaves (**Figures 7C,D**). H_2_O_2_ levels remained elevated for at least 48 h in both local and systemic leaves. After 72 h, no significant changes in H_2_O_2_ content were observed throughout the remaining period of the experiment. The accumulation of H_2_O_2_ was about 20 min earlier than that of NO in both local and systemic leaves, whilst the duration of high level H_2_O_2_ was much longer than that of NO. Besides, there exists a temporal overlap between wounding-induced NO and H_2_O_2_ from about 1 h to 6 h after wounding (**Figure 7**). To visually observe the signal transduction of H_2_O_2_, the vascular tissues were stained by H2DCF-DA fluorescent staining. **Figure 7E** shows that local mechanical wounding-induced H_2_O_2_ signal along the vascular tissue not only in local leaf and systemic leaf, but also in the sheaths of the leaves. The green fluorescent signals were significant in both leaf tissues and sheaths in wounded plants than their corresponding control.

The temporal changes in the activities of antioxidant enzymes were also investigated. In total, the activities of SOD, APX, and GR were increased concomitantly as early as at 3 h after mechanical wounding in both local and systemic leaves, whereas CAT activity was markedly elevated at 6 h in response to mechanical wounding (Supplementary Figure S3). The activities of all these antioxidant enzymes were peaked at 6 h in local leaves and at 12 h in systemic leaves. The elevated activities of these enzymes were persisted for about 1 to 2 days before declining to control levels after mechanical wounding. Moreover, the decline of these antioxidant activities was accompanied by the disappearance of the photosynthetic protection induced by mechanical wounding. These findings highlight the dominant roles of NO and H_2_O_2_ interrelating with the signaling responses to wounding and freezing.

### Identification and Expression Patterns of Freezing or/and Wounding-Induced Genes

**FIGURE 8:**
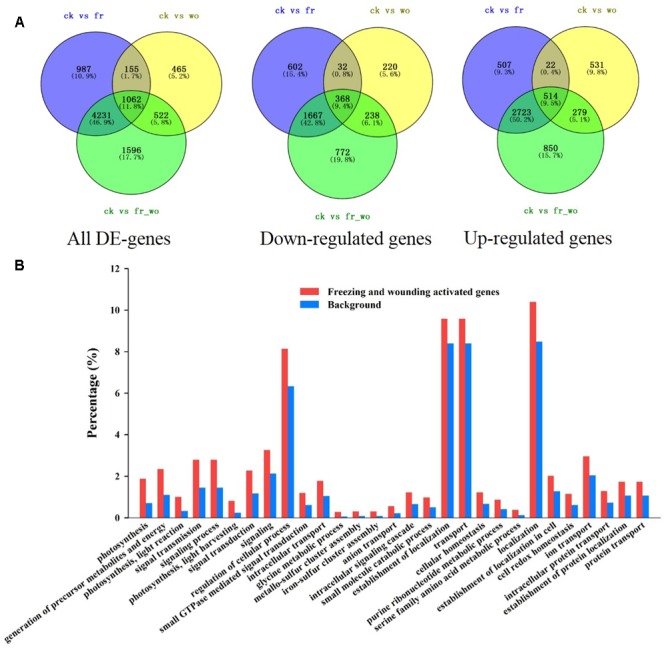
Transcriptional profiling of wheat plants due to the wounding and freezing treatment. Four treatments of systemic leaves: control (ck), 3 h after wounding (wo), 24 h after freezing (fr) and 3 h wounding + 24 h freezing (wo_fr) were collected for RNA sequencing analysis. The local (fourth) leaf was wounded prior to exposed to the freezing stress for 24 h. **(A)** Venn diagram illustrating the number of differentially expressed genes in plants treated with wounding, freezing, and wounding/freezing. The plants were treated as described in **Figure 1**; **(B)** GO analysis was performed for up-regulated genes in systemic leaves after wounding/freezing treatment.

To elucidate the molecular mechanisms underlying the wounding-mediated freezing tolerance in wheat, RNA-Seq assay was performed for systemic leaves after the following treatment: untreated (ck), wounding treatment for 3 h (wo), freezing treatment for 24 h (fr), and wounding for 3 h plus freezing treatment for 24 h (wo_fr). A total of 9018 different expressed genes (DE-genes) were detected compared with control leaves. Among them, 1062 genes were identified in the data from all three treatments, and specifically 514 were up-regulated while 368 were down-regulated in all three treatments (**Figure 8A** and Supplementary Figure S4). Comparing the up-regulated genes between freezing treatment and wounding/freezing treatment, 3237 genes were overlapped. However, 279 genes were only significantly up-regulated in the plants treatment with wounding and freezing, but not in the plants treated with freezing only, which implies that these 279 genes may contribute to wounding-induced freezing tolerance (**Figure 8A**). Among these 279 genes, 33 were involved in the categories “Photosynthesis” while eight genes were significantly increased in “ROS” (Supplementary Table S2).

In order to further understand the functions of wounding and freezing regulated genes, Gene Ontology (GO) enrichment analysis was performed for the 4264 differentially expressed genes after wounding and freezing treatment. Wounding and freezing regulated genes were significantly enriched in the categories “photosynthesis,” “generation of precursor metabolites and energy,” “photosynthesis, light reaction,” “signal transmission,” “signaling process,” “photosynthesis, light harvesting,” “signal transduction,” “signaling,” etc. (**Figure 8B**). Similarly, the differentially expressed genes in wounding plants were also enriched in “photosynthesis, light harvesting,” “photosynthesis,” etc. (Supplementary Figure S5B). In contrast, the differentially expressed genes in freezing plants were enriched in “signaling process,” “signal transmission,” etc., but not in “photosynthesis” (Supplementary Figure S5A). Altogether, the transcriptional profile supports our experimental data showing that pretreatment with wounding increases photosynthesis efficiency and ROS level of wheat under cold condition.

## Discussion

Over the years, a number of research groups have committed to reveal the alleviation effects of low temperature stress on plants at physiological and molecular levels (Li et al., 2013; Dong et al., 2014; Zhang et al., 2016). SAA, which is proposed as an effective method in response to various abiotic stresses, has been widely investigated (Capiati et al., 2006; Zhou et al., 2014; Mittler and Blumwald, 2015). In the present study, we have well demonstrated that mild mechanical wounding in local leaves is capable of enhancing subsequent freezing tolerance in systemic leaves of wheat plants. Local wounding treatment enhanced tolerance to freezing-induced photooxidative stress, considerably increased Pn and *F*v/*F*m (**Figure 1**), coupled with strengthened antioxidant system (**Figure 4** and Supplementary Figure S3) in untreated systemic leaves and ultimately alleviated freezing stress. Our research may contribute to develop novel management to mitigate the impact of low temperature stress and minimize the economic losses due to global climate change in wheat production.

### The Relationship between Wounding-Induced NO and H_2_O_2_

Systemic signaling has been demonstrated to play important roles in the process of SAA. It is well established that both NO and H_2_O_2_ take an active part in the signal cascade in response to mechanical wounding (Orozco-Cárdenas et al., 2001; Jih et al., 2003; Schilmiller and Howe, 2005; Arasimowicz et al., 2009). Wounding-induced H_2_O_2_ activated the expression of the defense-related gene *IPO* in sweet potato, identical to the inhibitor of NO, validating the antagonistic roles between NO and H_2_O_2_ in regulating wounding-induced defense system (Jih et al., 2003). However, a different picture emerged that NO donor acutely decreased H_2_O_2_ content after wounding in sweet potato, implying that NO acts in concert with H_2_O_2_ in response to mechanical wounding (Lin et al., 2011). The discrepancy between these studies implies that some unknown signaling pathways have yet to be elucidated. Therefore, the question of the relationship between H_2_O_2_ accumulation and NO production in wheat plants exposed to mechanical wounding appears to be particularly interesting.

In this paper, three lines of evidence illustrate that H_2_O_2_ acts upstream of NO in the wounding-induced antioxidant defense. First, the time-course analysis of H_2_O_2_ accumulation and NO production revealed that the generation of H_2_O_2_ preceded the production of NO in the wounding signal in both local and systemic leaves (**Figure 7**). Second, wounding-induced NO burst was nearly fully inhibited by pretreatment with H_2_O_2_ inhibitors or scavenger (**Figure 2**), suggesting that NO synthesis might be the result of H_2_O_2_ accumulation. Third, pretreatment with NO inhibitors and scavenger had little effects on wounding-induced H_2_O_2_ generation, indicating that NO synthesis is not required for the initial H_2_O_2_ accumulation (**Figure 2**). Taken together, our data suggest that wounding-induced H_2_O_2_ activates the systemic production of NO and eventually strengthened the antioxidant defense systems in wheat plants.

### Systemic Signal Transduction between NO and H_2_O_2_ in Response to Wounding

Evidences hints that NO is a short term signal which do not transport in long-distance in plant tissues (París et al., 2007; Mai et al., 2014). Instead, *S*-nitrosoglutathione (GSNO), formed by efficiently binding of NO to glutathione, has been proposed as a systemic wounding signal (Rustérucci et al., 2007; Espunya et al., 2012). As a relatively active molecule, however, H_2_O_2_ could migrate from the synthetic site to the neighboring vascular tissues or leaves (Buchanan and Balmer, 2005; Gilroy et al., 2014). Importantly, the systemic accumulation of H_2_O_2_ plays crucial roles in ameliorating various biotic and abiotic stimuli (Fryer et al., 2003; Bienert et al., 2006; Xia et al., 2011; Mignolet-Spruyt et al., 2016). In agreement with these findings, we observed that treatment with inhibitors and scavenger of NO in local leaves remarkably abolished wounding-induced NO accumulation in local leaves but had no influence on that in systemic leaves (**Figures 2A,D**). On the contrary, the inhibitor and scavenger of H_2_O_2_ sharply reduced H_2_O_2_ accumulation and $O_{2}^{–•}$ production rate in both local and systemic leaves (**Figures 2B–D**), raising the possibility that the accumulation of H_2_O_2_ in systemic leaves seem to result from local leaves along the path of the systemic signals while the systemic production of NO could be originated from H_2_O_2_ (**Figure 7**). Thus, it is necessary to investigate the possible source of H_2_O_2_ and NO in response to mechanical wounding.

Accumulating evidence postulated that wounding-induced RBOH is responsible for H_2_O_2_ accumulation (Sagi et al., 2004; Miller et al., 2009; Suzuki et al., 2012). In this work, when treatment of the local leaves with the RBOH inhibitor DPI or H_2_O_2_ scavenger DMTU prior to mechanical wounding, the concentration of H_2_O_2_ was dramatically decreased (**Figures 2B,D**). At subcellular level, DPI only scavenged the accumulation of H_2_O_2_ in the apoplast facing intercellular spaces but not those in chloroplasts (**Figure 6** and Supplementary Figure S1). Apparently, plasma membrane RBOH is the major but not the only source of H_2_O_2_ in response to mechanical wounding. There are hints suggesting that RBOH mediating H_2_O_2_ has also been demonstrated as the long-distance signal from stimulated parts to the whole plants in response to various abiotic stresses (Neill et al., 2002; Mittler et al., 2004, 2011). In agreement with these findings, H_2_O_2_ accumulation was only detected in the apoplast of the systemic leaf after wounding but not in chloroplasts (**Figure 6** and Supplementary Figure S2). To this regard, we confirm that RBOH in plasma membrane contribute to the rapid production of systemic H_2_O_2_ in the wounding signal cascade. NR and NOS have been implicated as two major enzymes responsible for NO biosynthesis in plants (Rockel et al., 2002; Besson-Bard et al., 2008; Corpas et al., 2008; Chaki et al., 2011). The possible source of the early burst NO involved in mechanical wounding was also detected and quantified in this paper by *in situ* and hemoglobin assay, respectively. NR inhibitor tungstate greatly abolished wounding-induced NO accumulation, a slight lower compared to that of L-NAME, a NOS inhibitor (**Figures 2A,D**). The activities of NR and NOS were further analyzed at 3 and 18 h after mechanical wounding as previously discussed (**Figure 5**), resulting in a conclusion that NR is the major source of early NO accumulation on local leaves in response to mechanical wounding.

### Crosstalk between H_2_O_2_ and NO in Wounding-Induced Freezing Tolerance

It has been repeatedly reported that both H_2_O_2_ and NO act as second messengers in perception and transduction of signals in alleviating low temperature stress (Prasad et al., 1994; Suzuki and Mittler, 2006; Esim and Atici, 2014). NO evoked by NR during cold acclimation alleviated the following freezing stress in Arabidopsis (Zhao et al., 2009); Coincidentally, H_2_O_2_ generated by RBOH played a vital role in cold acclimation-induced chilling tolerance (Zhou et al., 2012). Based on the previous results, we believe that the H_2_O_2_ and NO induced by mechanical wounding might contribute to the subsequent freezing tolerance. Thus, a hypothesis has been put forward that local mechanical wounding-induced H_2_O_2_ and NO in local leaves, triggered the systemic signals along the pathway, and subsequently regenerated H_2_O_2_ together with NO in systemic leaves, which in turn lead to the up-regulation of antioxidant defense systems in wheat plants. Our results revealed the distinctive roles of mechanical wounding in strengthening freezing tolerance via the process of H_2_O_2_ and NO accumulation. We first observed that NO was accumulated not only in wounded local leaves, but also in untreated systemic leaves of wheat plants (**Figure 7**). Pretreatment of the NO synthetic inhibitors L-NAME and tungstate or NO scavenger c-PTIO substantially down-regulated the activities of antioxidant enzymes and consequently reduced tolerance to freezing stress in systemic leaves (**Figures 3, 4**). Additionally, the strengthened antioxidant system in response to mechanical wounding were consistent with the temporal changes of wounding-induced H_2_O_2_ in both local leaves and systemic leaves (Supplementary Figure S3). Besides, the protection mechanism of mechanical wounding on photosystem sustained for at least 3 days compared to that of SNP pretreatment (Supplementary Figure S1). One potential explanation cannot be discarded because that other signal molecules or phytohormones instead of H_2_O_2_ and NO are involved in the resistance of freezing stress such as ABA, salicylic acid, jasmonic acid, and polyamines (Shen et al., 2000; Dong et al., 2014; Wang et al., 2016) which have yet to be fully elucidated in future studies. Taken together, the sustained freezing tolerance in wounded plants is likely to be mediated by the systemic accumulation of NO in a H_2_O_2_ dependent manner, namely, H_2_O_2_ and NO act sequentially and synergistically in the early phase of this cross-tolerance signaling network.

### Wounding-Induced Freezing Tolerance by Enhancing Antioxidant System and Photosystem via ROS Signal

Under freezing conditions, ROS may play two different roles: exacerbating the damage, or signaling the activation of defense responses (Baxter et al., 2014). Here, the accumulated H_2_O_2_ in systemic leaves after mechanical wounding could play positive roles in freezing tolerance other than causing oxidation of cellular components. Although the concentration of H_2_O_2_ in systemic leaves was increased upon wounding (**Figure 7C**), the activities of antioxidant enzymes were increased as well, which may contribute to alleviating oxidative stress (Supplementary Figure S3). On the other hand, the decreased REC and MDA content were also observed (**Figure 1B**), thus validating the regulatory role of H_2_O_2_ in antioxidant system in wounding-induced freezing tolerance.

Literatures indicate that wounding indirectly suppressed photosynthesis of remaining undamaged leaves (Zangerl et al., 2002; Aldea et al., 2005; Nabity et al., 2009). In this paper, however, the ameliorating effects of mechanical wounding on photosystem under freezing stress were observed at both physiological and transcriptional levels, thus validating the dual role of mechanical wounding on photosystem. The strong arguments between the previous studies and ours may due to three main reasons. First, in stances they use arthropod herbivory as wounding treatment tools while we use hemostat which may results in different kinds of initiations of wounding signal. Second, we use wheat, a kind of monocotyledon as plant material in which the vascular tissues are unique from those in dicotyledons. The systemic tissues analyzed in previous studies were connected to wounded leaves or even in the same leaf. In our study, however, the wounding signal ROS were detected in sheaths of both local and systemic leaves (**Figure 7E**), indicating a more complicated signal transduction mechanism in monocotyledons. Third, previous reporters focused on the systemic changes of photosystem after wounding treatment alone, whereas we not only interpreted the essential of wounding signal, but also shed light on the alleviation effects of mechanical wounding on freezing stress. At first glance, wounding suppressed photosynthesis of systemic leaves, however, the reinforcement of photosystem was emerged after exposed to freezing stress. The potential explanation of this phenomenon is that wounding-induced systemic ROS signal enhanced the photosystem thus contributed to the acquisition of freezing tolerance (**Figures 1, 3, 4, 8**).

## Conclusion

**FIGURE 9:**
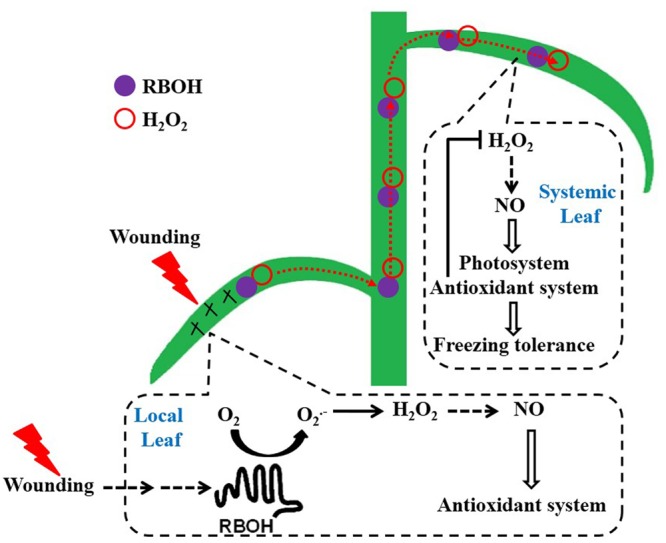
The working model illustrating the induction of freezing tolerance by mechanical wounding in wheat plant. Perception of wounding signal in local leaf results in the accumulation of H_2_O_2_ systemically (resulted from the plasma membrane-bound NADPH oxidase). Elevated levels of H_2_O_2_ triggers and amplifies the signaling pathway of wounding and then induces NO in a NR-dependent manner in systemic leaf. NO further functions as a signal molecule to up-regulate the photosystem and antioxidant system in response to freezing stress.

We have presented strong physiological, biochemical, and transcriptional experiments that local mechanical wounding induces early NO burst through a NR route in a H_2_O_2_-dependent manner (mediated by plasma membrane RBOH). Then, H_2_O_2_ triggers and amplifies the signaling pathway of wounding to systemic leaves within minutes and reproduced NO for signaling purpose. H_2_O_2_ and NO then act in concert and ultimately strengthen the photosystem as well as the antioxidant defense system to alleviate oxidative states arising from freezing stress in systemic leaves (**Figure 9**). To the best of our knowledge, it was the first time to reveal this new type of SAA, i.e., alleviation effects of mechanical wounding on freezing tolerance in wheat. Additional work using proteomic and molecular approaches are required to understand the detailed mechanism of wounding-induced freezing tolerance.

## Author Contributions

TS and DJ conceived and designed the experiments; TS, XW, LW, LZ, MH, JC, QZ, and TD performed the experiments; TS, J-KZ, and CZ analyzed the RNA-seq data; TS and DJ wrote the article with contribution of all the authors.

## Conflict of Interest Statement

The authors declare that the research was conducted in the absence of any commercial or financial relationships that could be construed as a potential conflict of interest.

## Footnotes

1 https://www.r-project.org/
