# Supplementary material for: Nitric Oxide and Hydrogen Peroxide Mediate Wounding-Induced Freezing Tolerance through Modifications in Photosystem and Antioxidant System in Wheat
Source: Front Plant Sci. 2017 Jul 19;8:1284. doi: 10.3389/fpls.2017.01284 (PMC5515872; doi:10.3389/fpls.2017.01284)
Supplement: Supplementary file 1 [file Data_Sheet_1.DOCX]

Supplementary Material

**Title:** Nitric oxide and hydrogen peroxide mediate wounding induced freezing tolerance through modifications in photosystem and antioxidant system in wheat

**Authors:** Tong Si^1,2^, Xiao Wang^1^, Lin Wu^1^, Chunzhao Zhao^2^, Lini Zhang^1^, Mei Huang^1^, Jian Cai^1 *^, Qin Zhou^1^, Tingbo Dai^1^, Jian-Kang Zhu^2^, Dong Jiang^1 **^

**^*^Co-correspondence author:** Jian Cai: caijian@njau.edu.cn

**^**^Correspondence author:** Dong Jiang: jiangd@njau.edu.cn

**
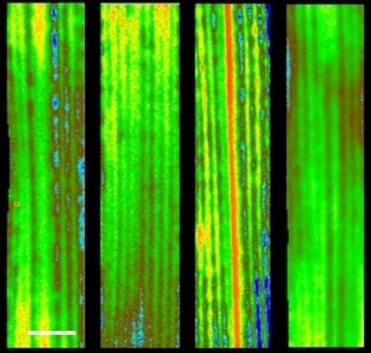

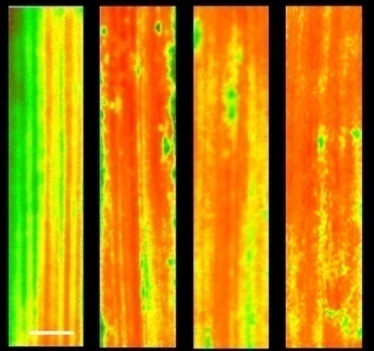
**

**Wounding +Freezing**

**SNP +Freezing**

**Water + Freezing**

**
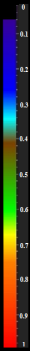

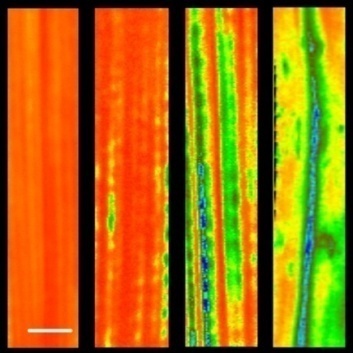
**

**3h 12h 24h 72h**

**3h 12h 24h 72h**

**3h 12h 24h 72h**

**Supplementary Figure S1** Dynamic changes in Fv/Fm of the system leaf after mechanical wounding and SNP treatment. The local (fourth) leaf was pretreated with distilled water (squares), 200 µM SNP (circles) or wounded (triangles). Freezing stress was applied to all the plants at indicated times after these treatments and Fv/Fm was determined by the systemic (fifth) leaf as area of interest after 24 h freezing stress. Time zero points indicate freezing treatment only. Data are means ± SD of three different replicates. Representative imagines of Fv/Fm were also shown at 3, 12, 24 and 72 h after water, 200 µM SNP or wounding treatment, respectively. The horizontal false color code ranges from 0.0 (black) to 1.0 (red). Bars, 10mm.

**
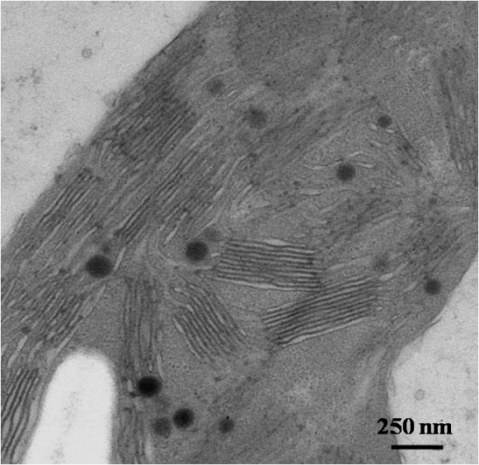

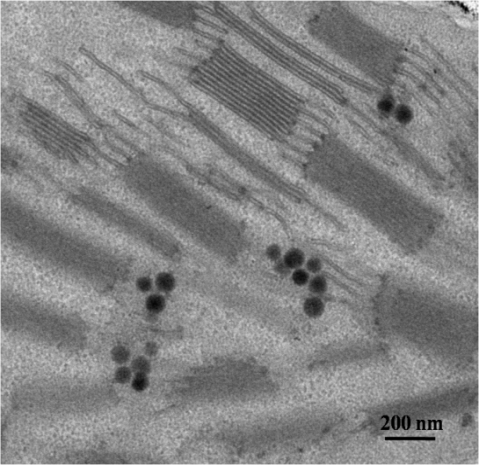
Local leaf Systemic leaf**

**Control**

**
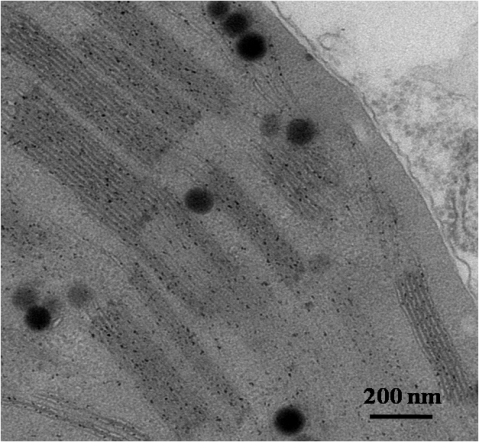

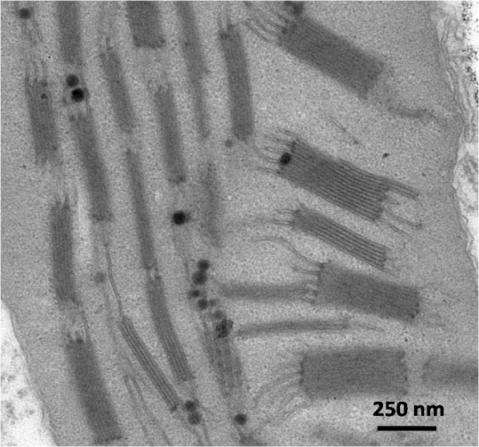
**

**Wounding**

**
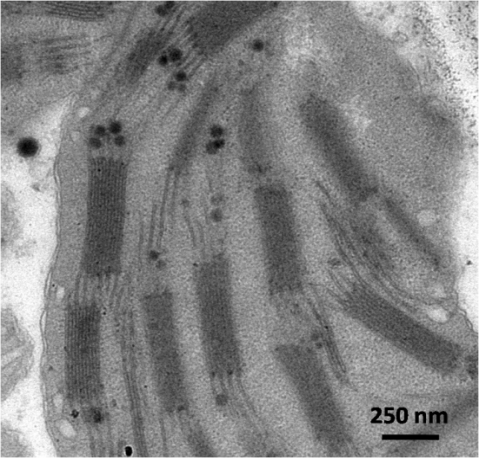

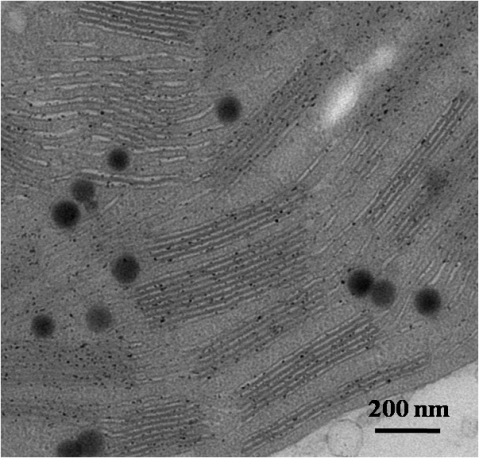
**

**DPI+**

**Wounding**

**Supplementary Figure S2** The cytochemical localization of H_2_O_2_ in local and systemic leaves in chloroplast. The local leaf was pretreated with DPI or distilled water as control. Mechanical wounding was conducted 8 h later. 12 h after wounding, they were used for CeCl_3_ staining and detected by a transmission electron microscopy. Arrows, CeCl_3_ precipitates; C, chloroplast; CW, cell wall; IS, intercellular space; V, vacuole.

**Supplementary Figure S3** Time-course analysis of antioxidant enzymes activities in response to mechanical wounding. The local leaves were prior wounded, then the local (fourth) and systemic (fifth) leaves of wounded (circles) and untreated (squares) plants were harvested at indicated times (h) after wounding treatment to determine the activities of antioxidant enzymes. Data are means ± SD of three different replicates.


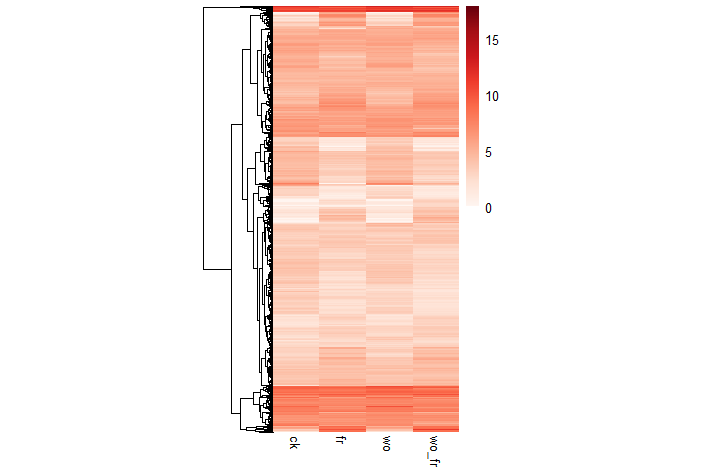


**Supplementary Figure S4** Hierarchical clustering of genes that were up-regulated or down-regulated in different treatments. Four treatments of systemic (fifth) leaves: control (ck), 3 h after wounding (wo), 24 h after freezing (fr) and 3 h wounding + 24 h freezing (wo_fr) were collected for hierarchical clustering analysis. The local (fourth) leaf was wounded prior to exposed to the freezing stress for 24 h.

**
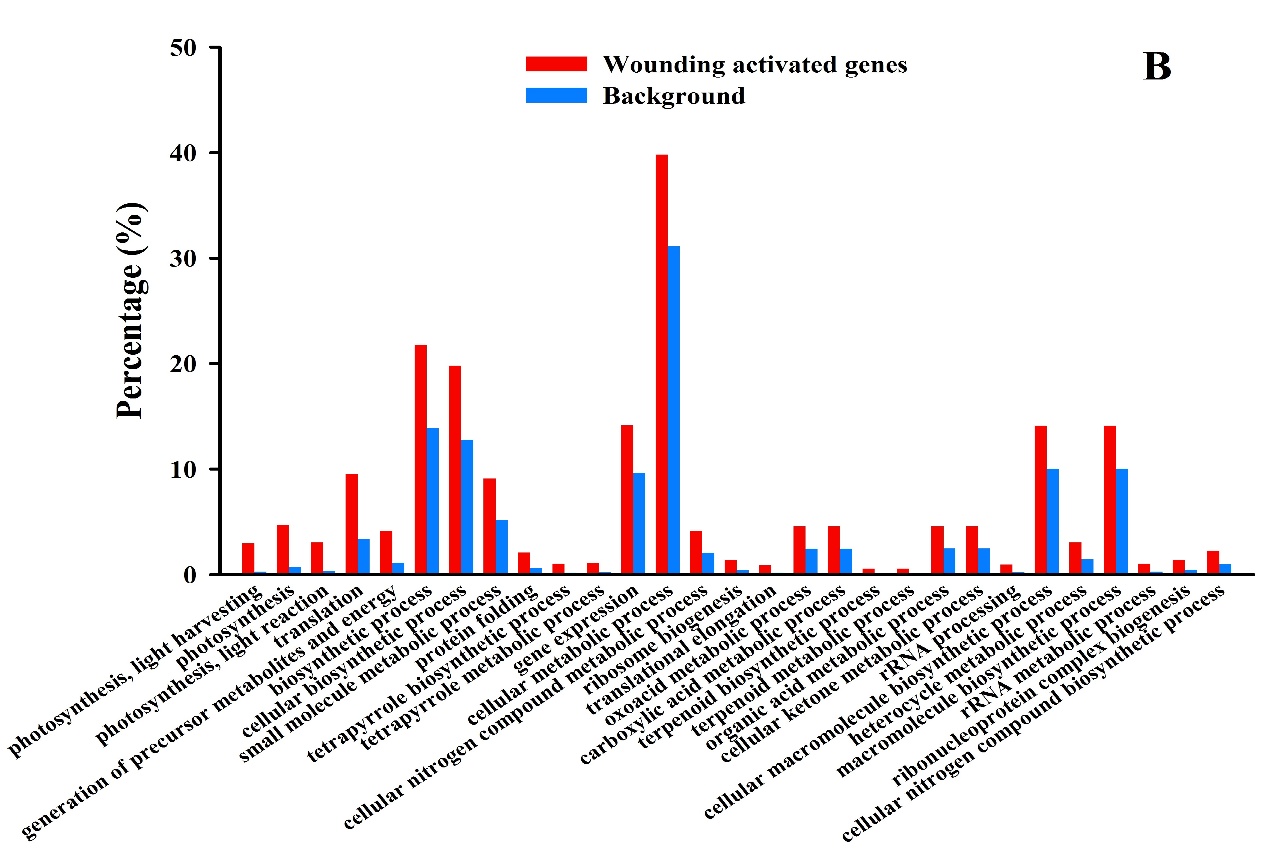

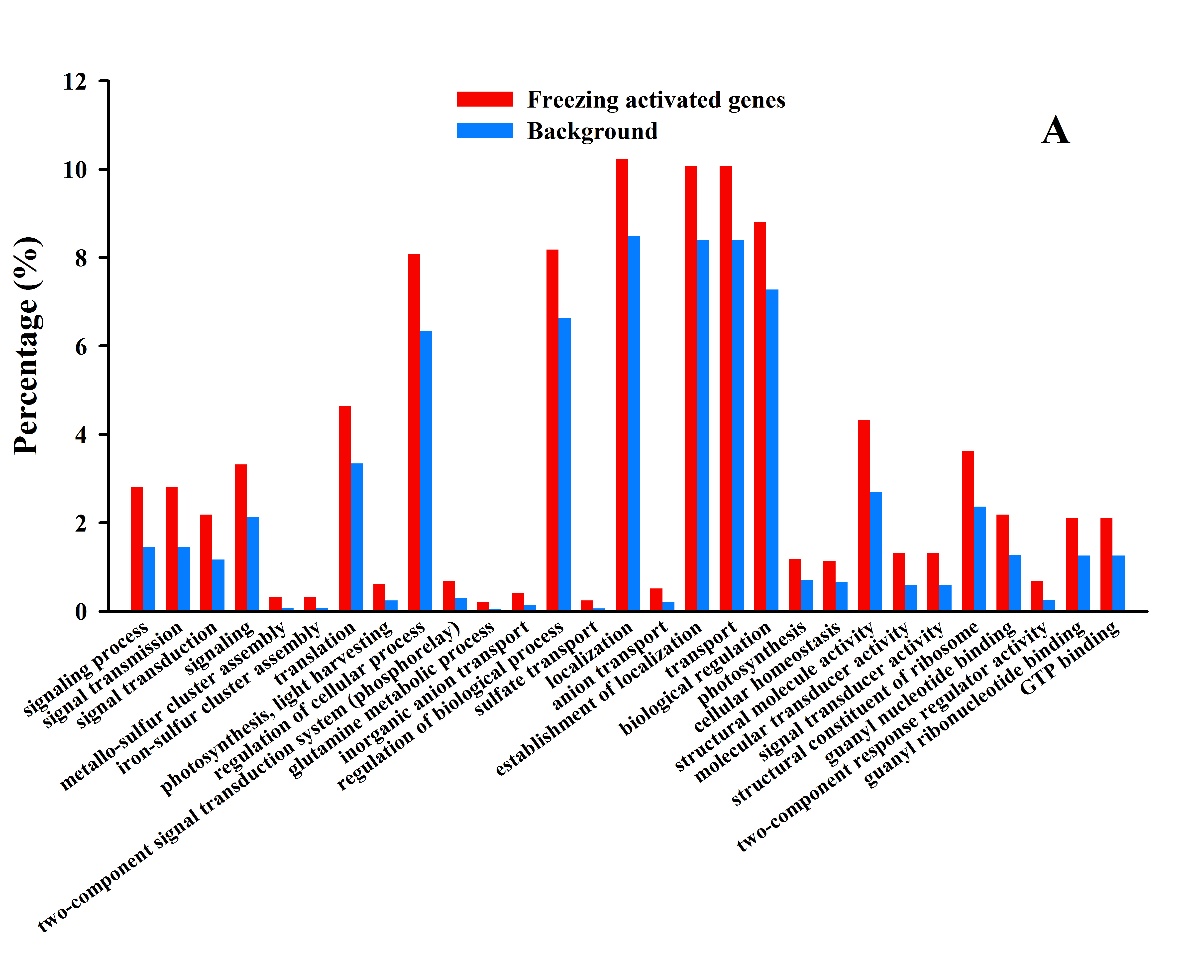
Supplementary Figure S5** GO enrichment analysis of the number and overlap of activated genes in different treatments. Two treatments of systemic (fifth) leaves: 24 h after freezing (fr) (A) and 3 h after wounding (wo) (B) were collected for GO enrichment analysis. The local (fourth) leaf was wounded prior to exposed to the freezing stress for 24 h.

**Supplementary Table S1** Gene-specific primers designed for RT-PCR.

| Gene | Accession | Forward sequence (5’-3’) | Reverse sequence (5’-3’) |
| --- | --- | --- | --- |
| *Actin* | AB181991 | GCTCGACTCTGGTGATGGTG | AGCAAGGTCCAAACGAAGGA |
| *SOD* | AF092524 | CCGGACTACCTGACCAACATC | CCAACAGCGGGAAACTCAA |
| *CAT* | D86327 | CCATGAGATCAAGGCCATCT | ATCTTACATGCTCGGCTTGG |
| *APX* | U15933 | GCAGCTGCTGAAGGAGAAGT | CACTGGGGCCACTCACTAAT |
| *GR* | AY364467 | TGCGTCCCGAAGAAGATACT | GTTGATGTCCCCGTTGATCT |
| *RBOH* | AY561153 | ATGTTCGGCAACTTGGTGACT | CGTCTGCTCTAAGAAGACCACTTTT |

| **Supplementary Table S2** Biological functions analysis of the differentially expressed genes. Predicted up-regulated genes and target annotation in photosynthesis and reactive oxygen species compared with freezing treatment and wounding/freezing treatment. Only one of some common protein (family) was listed. | | | | |
| --- | --- | --- | --- | --- |
| **Predicted target genes** | **value_1** | **value_2** | **log2(fold_change)** | **Target annotation** |
| **Photosynthesis** |  |  |  |  |
| \| Traes_7BS_91EA4C6E5 \| \| --- \| \| Traes_7BS_6606D4150 \| \| Traes_7AS_D2C7C1F43 \| \| Traes_7AS_0A1BAEEAD \| \| Traes_6DS_89CCF9552 \| \| Traes_6DS_6FEF78A19 \| \| Traes_6DS_51B66DB37 \| \| Traes_6AS_F8D06BEC8 \| \| Traes_6AS_E5254BC5B \| \| Traes_6AS_58D403D66 \| \| Traes_5DL_811A8382F \| \| Traes_5DL_6E360A62C \| \| Traes_5DL_60C61B6C0 \| \| Traes_5DL_1F44EFC2C \| \| Traes_5BL_E0E94A9A9 \| \| Traes_5BL_6FB7A4B58 \| \| Traes_5AL_9BB3D36D2 \| \| Traes_5AL_3AF3E7C87 \| \| Traes_5AL_16F3640B6 \| \| Traes_4BS_A666283DF \| \| Traes_4AL_84CD4FDBD \| \| Traes_2DS_F7D244C5A \| \| Traes_2BL_7E386E5FA \| \| Traes_2AS_6B8CE04FD \| **value_1** \| **value_2** \| **log2(fold_change)** \| **Target annotation** \| \| Traes_2AL_E7B360A43 \| \| Traes_1DL_A6E0B4612 \| \| Traes_1DL_18BBECFD8 \| \| Traes_1BL_E7A59AA9C \| \| Traes_1BL_E38BBC0DA1 \| \| Traes_1BL_D2EDF576B \| \| Traes_1BL_6CCAC095C \| \| Traes_1AL_B77FFBAFC \| \| Traes_1AL_6E5BA9C6F \| \| **Reactive oxygen species**  Traes_2BL_E6F86DAFA  Traes_6AL_80FD46553  Traes_2AL_C6F2FA817  Traes_2DL_4AA8E1AA9  Traes_2AL_7EABAC855  Traes_6AS_BE4F761EB1  Traes_2BL_E3222439E  Traes_6DS_3522B8EF6 \| | 128.505  58.3449  81.9531  3994.95  13.2729  16.7273  88.902  270.025  258.543  158.577  5.59855  58.2053  424.934  90.3413  101.997  321.573  198.566  123.091  189.127  2565.56  2932.75  8555.1  1280.96  676.623  1776.21  110.462  35.5601  249.663  205.7  236.828  217.352  167.926  114.176  387.427  415.953  278.485  146.527  135.366  52.6426  143.471  1.36942 | 197.22  107.573  182.955  9584.3  28.5507  32.5603  192.267  564.011  585.121  366.723  12.9526  100.326  843.708  186.455  189.095  700.897  344.59  237.611  459.254  4066.43  4790.51  15483.3  2243.35  1300.08  3049.06  192.284  59.5124  613.794  566.117  479.198  418.779  406.1  266.135  572.782  622.554  421.387  221.833  206.317  90.6755  281.579  5.24129 | \| 0.617977 \| \| --- \| \| 0.882642 \| \| 1.15862 \| \| 1.26249 \| \| 1.10504 \| \| 0.960911 \| \| 1.11283 \| \| 1.06263 \| \| 1.17833 \| \| 1.20951 \| \| 1.21012 \| \| 0.785477 \| \| 0.989505 \| \| 1.04537 \| \| 0.890581 \| \| 1.12406 \| \| 0.795261 \| \| 0.948874 \| \| 1.27994 \| \| 0.664492 \| \| 0.707927 \| \| 0.855858 \| \| 0.808433 \| \| 0.942173 \| \| 0.779565 \| \| 0.799691 \| \| 0.742929 \| \| 1.29777 \| \| 1.46056 \| \| 1.01678 \| \| 0.946155 \| \| 1.27401 \| \| 1.22089 \|   0.564062  0.58178  0.597549  0.598305  0.608003  0.784481  0.972783  1.93636 | \| protein 1C,chlorophyll binding \| \| --- \| \| Chlorophyll a-b binding protein,chloroplastic \| \| Chlorophyll a-b binding protein,chloroplastic \| \| Chlorophyll a-b binding protein 1B,chloroplastic \| \| chlorophyll a/b-binding protein WCAB precursor \| \| chloroplast light-harvesting chlorophyll a/b binding protein \| \| chlorophyll a/b-binding protein WCAB precursor \| \| chlorophyll a/b-binding protein WCAB precursor \| \| Unknown \| \| chlorophyll a/b-binding protein WCAB precursor \| \| PREDICTED: chlorophyll a-b binding protein of LHCII type 1 \| \| PREDICTED: chlorophyll a-b binding protein 7, chloroplastic \| \| chloroplast light-harvesting chlorophyll a/b binding protein \| \| chlorophyll a/b-binding protein WCAB precursor \| \| Unknown \| \| chlorophyll a/b-binding protein WCAB precursor \| \| chlorophyll a/b-binding protein WCAB precursor \| \| chlorophyll a/b-binding protein WCAB precursor \| \| chlorophyll a/b-binding protein WCAB precursor \| \| Precursor of CP29, core chlorophyll a/b binding (CAB) protein of  photosystem II (PSII) \| \| Chlorophyll a-b binding protein CP26, chloroplastic \| \| PREDICTED: chlorophyll a-b binding protein 7, chloroplastic \| \| PREDICTED: chlorophyll a-b binding protein CP24, chloroplastic \| \| Chlorophyll a-b binding protein CP29.2, chloroplastic \| \| PREDICTED: chlorophyll a-b binding protein CP24, chloroplastic \| \| Chlorophyll a-b binding protein 3C, chloroplastic \| \| chloroplast light-harvesting chlorophyll a/b binding protein \| \| chloroplast light-harvesting chlorophyll a/b binding protein \| \| chloroplast light-harvesting chlorophyll a/b binding protein \| \| chlorophyll a/b-binding protein WCAB precursor \| \| chlorophyll a/b-binding protein WCAB precursor \| \| chlorophyll a/b-binding protein WCAB precursor \| \| Unknown \|   2-cys peroxiredoxin BAS1,chloroplastic  thylakoid bound ascorbate peroxidase  2-cys peroxiredoxin BAS1  2-cys peroxiredoxin BAS1  multiple organellar RNA editing factor 2,chloroplastic  peroxiredoxin-2E-2  thylakoid lumenal 29 kDa protein  Catalase isozyme 2 |
